# Australian best practice recommendations for transjugular intrahepatic portosystemic shunt (TIPS) in portal hypertension: a consensus statement

**DOI:** 10.1007/s12072-025-11023-x

**Published:** 2026-03-03

**Authors:** Eric Kalo, Jacinta Holmes, Purnima Bhat, Winita Hardikar, Nishita Jagarlamudi, Wai See Ma, Cositha Santhakumar, Scott Read, Alicia Braund, Fei Wen Chen, Rohit Gupta, Kate Collins, Anouk Dev, John Grieve, Jim Koukounaras, Shivendra Lalloo, Vi Nguyen, Tim Mitchell, Nigel Mott, Ashok Raj, Dinesh Ranatunga, Caroline Tallis, Edmund Tse, Zina Valaydon, Marcus Robertson, Diederick De Boo, Enoka Gonsalkorala, Rashid Muddassir, Thao Lam, Jonathan Tibballs, Jonathan Mitchell, Penny Fox, Adam Doyle, Rozemary Karamatic, Gerry Macquillan, Christopher Rogan, Jacob George, Nick Shackel, Stuart Roberts, John Olynyk, Barbara Leggett, Siddharth Sood, Simone Strasser, Radha Popuri, John Lubel, Miriam Levy, Jane Li, James O’Beirne, Avik Majumdar, Golo Ahlenstiel, Gastroenterological Society of Australia (GESA)

**Affiliations:** 1 College of Medicine and Dentistry, James Cook University, Smithfield, QLD Australia; 2 Blacktown Mt Druitt Clinical School, Western Sydney University, Blacktown, NSW Australia; 3 Department of Gastroenterology and Hepatology, St Vincent’s Hospital Melbourne, Fitzroy, NSW, Australia; 4 Faculty of Medicine, University of Melbourne, Parkville, VIC Australia; 5 Department of Gastroenterology and Hepatology, Canberra Hospital, Canberra, ACT, Australia; 6 College of Health and Medicine, Australian National University, Canberra, ACT Australia; 7 Department of Gastroenterology, Hepatology and Clinical Nutrition, Royal Children’s Hospital, University of Melbourne, Parkville, VIC Australia; 8 Department of Gastroenterology and Hepatology, Blacktown Hospital, Blacktown, NSW, Australia; 9 Department of Gastroenterology and Hepatology, Gold Coast University Hospital, Southport, QLD Australia; 10 Department of Gastroenterology and Hepatology, St George Hospital, Kogarah, NSW Australia; 11 Department of Gastroenterology and Hepatology, Nambour General Hospital, Nambour, QLD, Australia; 12 School of Medicine, University of the Sunshine Coast, Maroochydore, QLD Australia; 13 Department of Gastroenterology and Hepatology, Monash Medical Centre, Clayton, VIC, Australia; 14 Department of Medicine, Monash University, Clayton, VIC Australia; 15 Department of Radiology-Interventional, Alfred Hospital, Melbourne, VIC, Australia; 16 Central Clinical School, Monash University, Melbourne, VIC Australia; 17 Department of Gastroenterology and Hepatology, Royal North Shore Hospital, St Leonards, NSW, Australia; 18 Northern Clinical School, University of Sydney, St Leonards, NSW Australia; 19 Department of Gastroenterology and Hepatology, Royal Perth Hospital, Perth, WA, Australia; 20 School of Medicine, University of Western Australia, Perth, WA Australia; 21 Department of Gastroenterology and Hepatology, Royal Brisbane and Women’s Hospital, Herston, QLD, Australia; 22 School of Medicine, University of Queensland, Brisbane, QLD Australia; 23 Department of Gastroenterology and Hepatology, Royal Melbourne Hospital, Parkville, VIC, Australia; 24 Department of Medicine, University of Melbourne, Parkville, VIC Australia; 25 Department of Gastroenterology and Hepatology, Princess Alexandra Hospital, Brisbane, QLD, Australia; 26 Department of Gastroenterology and Hepatology, Royal Adelaide Hospital, Adelaide, SA, Australia; 27 Discipline of Medicine, University of Adelaide, Adelaide, SA Australia; 28 Department of Gastroenterology and Hepatology, Western Health, Melbourne, VIC, Australia; 29 Department of Medicine, University of Melbourne, Footscray, VIC Australia; 30 Department of Gastroenterology and Hepatology, Sir Charles Gairdner Hospital, Nedlands, WA, Australia; 31 Department of Gastroenterology and Hepatology, Cairns Hospital, Cairns, QLD, Australia; 32 Cairns Clinical School, James Cook University, Cairns, QLD Australia; 33 Faculty of Nursing and Midwifery, Sunshine Coast Hospital and Health Service, Birtinya, QLD, Australia; 34 University of the Sunshine Coast, Maroochydore, QLD Australia; 35 Department of Gastroenterology and Hepatology, Townsville Hospital and Health Service, Douglas, QLD, Australia; 36 College of Medicine and Dentistry, James Cook University, Townsville, QLD Australia; 37 Department of Gastroenterology and Hepatology, Westmead Hospital, Westmead, NSW, Australia; 38 Faculty of Medicine and Health, University of Sydney, St Leonards, NSW Australia; 39 Department of Gastroenterology and Hepatology, Launceston General Hospital, Launceston, TAS, Australia; 40 University of Tasmania, Launceston, TAS Australia; 41 Department of Gastroenterology, Fiona Stanley Hospital, Murdoch, WA Australia; 42 School of Medical and Health Sciences, Edith Cowan University, Joondalup, WA Australia; 43 Department of Gastroenterology and Hepatology, The Northern Hospital, Northern Health, Epping, Melbourne, VIC Australia; 44 Morrow Gastroenterology and Liver Centre Royal Prince Alfred Hospital, and University of Sydney, Sydney, NSW Australia; 45 Department of Gastroenterology and Hepatology, Liverpool Hospital, Liverpool, NSW, Australia; 46 Southwestern Sydney Clinical School, University of New South Wales, Liverpool, NSW Australia; 47 Department of Gastroenterology and Hepatology, Sunshine Coast University Hospital, Birtinya, QLD, Australia; 48 School of Medicine, University of the Sunshine Coast, Sippy Downs, QLD Australia; 49 Victorian Liver Transplant Unit, Austin Health, Heidelberg, VIC Australia; 50 Gastroenterological Society of Australia (GESA), Level 3, 517 Flinders Lane, Melbourne, VIC 3000 Australia

**Keywords:** TIPS, Australia, Consensus, Portal hypertension, Cirrhosis

## Abstract

**Background:**

Transjugular intrahepatic portosystemic shunt (TIPS) is one of the preferred interventional radiology techniques for reducing clinically significant portal pressures in patients with cirrhosis and complications from portal hypertension when pharmacological therapy or endoscopic interventions have failed or been insufficient. Recent advances in TIPS procedural techniques and stent technology, along with emerging indications for TIPS, warrant a review of current practices and establishment of consensus recommendations in Australia, where TIPS remains underused. This TIPS consensus statement is the first such guideline in Australia. It outlines 69 evidence-based practice recommendations and the evidence underlying them. The recommendations are intended for use by health care professionals in Australia who manage adult patients with portal hypertensive complications of liver disease, where such patients are being considered for TIPS implantation, including pre-, peri- and post-procedural aspects of care.

**Methods and results:**

This consensus statement has been developed by specialists in hepatology and interventional radiology, with input from specialists in cardiology, hematology and primary care, including medical practitioners, nurses and clinical researchers. The statement deals with four domains related to TIPS: preparation for TIPS, patient selection and pre-TIPS workup; best procedural practice; postoperative care and follow-up; and indications for TIPS. Two rounds of a modified Delphi process were used to reach consensus on the recommendations.

**Conclusions:**

Adoption of and adherence to the evidence-based recommendations in this consensus statement should reduce clinical variation. Ultimately, this should lead to system-level improvements in quality of care and outcomes for patients undergoing TIPS implantation. These recommendations summarize the complete document, available at https://www.gesa.org.au/resources/.

## Introduction

Transjugular intrahepatic portosystemic shunt (TIPS) is one of the most effective therapeutic options for treating complications of portal hypertension. Guidance on TIPS has been published by numerous international groups, including the American Association for the Study of Liver Diseases (AASLD) in 2005 (with updates in 2009 and 2023), the European Association for the Study of the Liver (EASL) in 2018 and the British Society of Gastroenterology in 2020, along with the North American practice-based recommendations for TIPS from the Advancing Liver Therapeutic Approaches (ALTA) Consortium in 2021 and the Baveno VII recommendations of 2022 [1–11]. Moreover, recent advances relating to novel procedural techniques, TIPS stent technology and emerging indications for TIPS have contributed significantly to the body of evidence for the effectiveness and safety of TIPS.

With this rapid evolution of knowledge, there remains a lack of consensus about which patients should receive a TIPS and an absence of dedicated guidance on TIPS referral pathways and practice guidelines in the Australian context. Consequently, Australian institutions demonstrate marked variability in practice and outcomes, with critical knowledge gaps revealed by a recent national survey of TIPS centers [12]. One of the key aims of guideline development is to promote dialogue among and between referring teams, multidisciplinary teams at TIPS centers and proceduralists.

## Methods

**Fig. 1:**
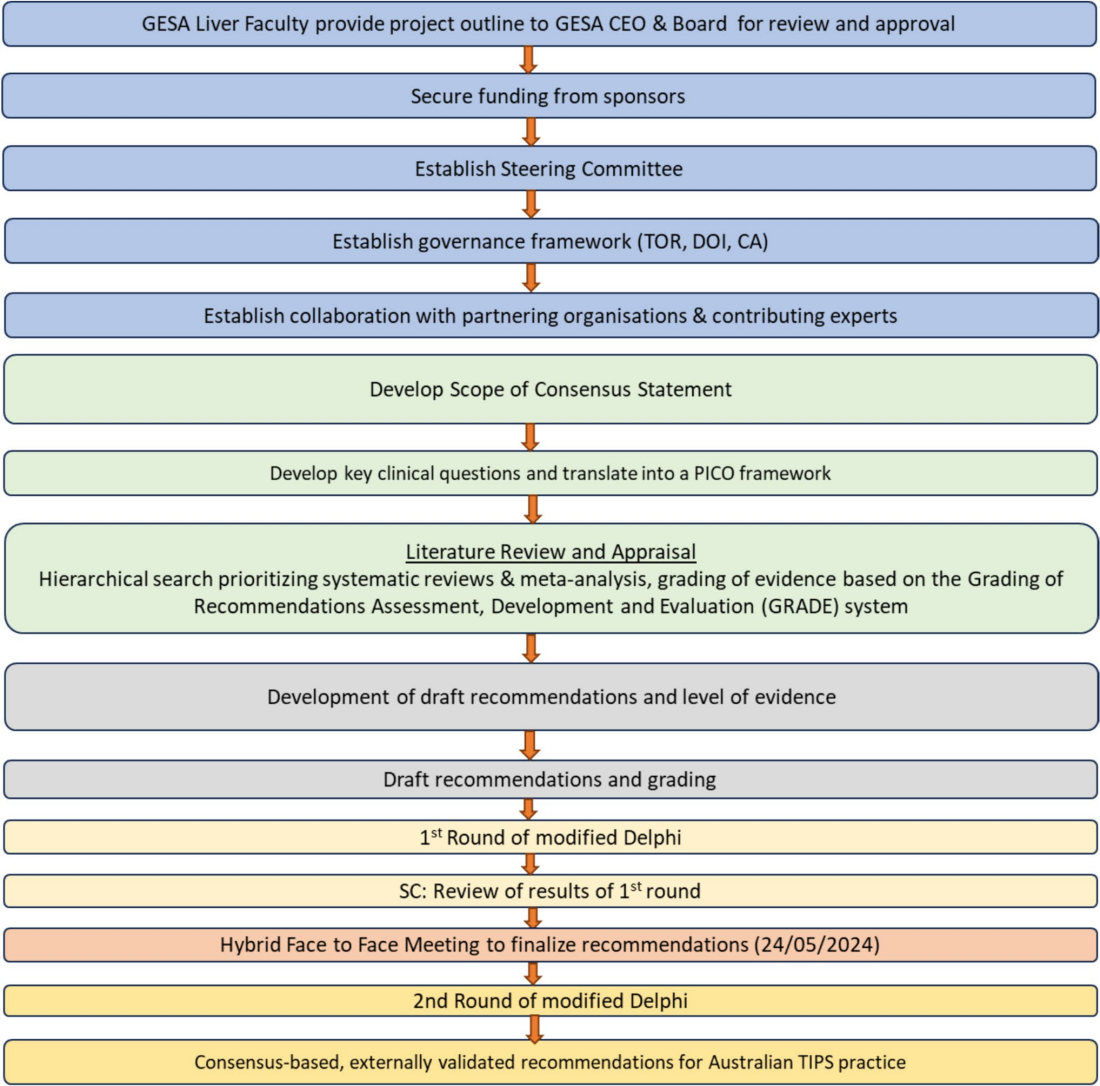
Methodological diagram outlining the stepwise m-Delphi process leading to final Consensus statements. CA: Collaboration Agreement; CEO: Chief Executive Officer; DOI: Declaration of Interests; GESA: Gastroenterological Society of Australia; GRADE: Grading of Recommendations, Assessment, Development and Evaluation; m-Delphi: Modified Delphi; PICO: Population, Intervention, Comparison, Outcome; SC: Steering Committee; TIPS: Transjugular Intrahepatic Portosystemic Shunt; TOR: Terms of Reference

The development of the consensus statement comprised elected members of the GESA Liver Faculty, representatives from IRSA and individuals from all relevant professional groups, including specialist clinicians, nurses and researchers with expertise in management of portal hypertension-related complications and the use of TIPS. A modified Delphi (*m*-Delphi) approach is used to achieve consensus (Fig. 1). A consensus process was conducted in accordance with the standards outlined in the Appraisal of Guidelines for Research and Evaluation II (AGREE II) [13].

Evidence supporting each recommendation was derived from a structured review of English-language literature published between January 1990 and June 2023, using PubMed, MEDLINE, EMBASE, Web of Knowledge, and the Cochrane Database. Searches combined core keywords with topic-specific terms relevant to each clinical domain. Evidence was appraised hierarchically, prioritizing randomized controlled trials, followed by meta-analyses, systematic reviews, and observational studies; high-quality case reports and existing evidence-based guidelines were considered where data were limited. Systematic reviews were assessed using the AMSTAR tool, and the quality and strength of evidence were graded according to the GRADE framework, consistent with the NHMRC 2016 standards for guideline development. Draft statements were synthesized through iterative expert discussion to ensure evidence alignment and clinical relevance.

The level of evidence for all consensus statements was appraised according to Grading of Recommendations Assessment, Development and Evaluation (GRADE) system, as recommended by the National Health and Medical Research Council Guidelines for Guidelines [14, 15]. The quality of the evidence was classified as high, moderate, low, or very low, and the strength of recommendation was classified as either strong or weak (Table 1).

**Table 1:** Summary of recommendations

Recommendation		Quality of evidence	Strength of recommendation
**Preparation for TIPS, patient selection and pre-TIPS workup**			
*Preparation for TIPS*			
1.1	The decision to proceed with TIPS implantation should be made by a multidisciplinary team	Low	Strong
1.2	TIPS implantation should be performed by an appropriately trained interventional radiologist who is a member of the TIPS multidisciplinary team	Moderate	Strong
1.3	TIPS implantation should occur at centers with relevant experience in performing the procedure and specialist services available for managing complications	Low	Strong
*Patient selection*			
2.1	Prognostic scoring systems for patients with cirrhosis should inform clinical decision-making	Moderate	Strong
2.2	Patients with an estimated high risk of reduced survival after TIPS implantation should be identified using prognostic scores during assessment	Moderate	Strong
2.3	Universal evaluation for liver transplantation before elective TIPS implantation is not mandatory	Low	Strong
*Pre-TIPS workup*			
(a) Imaging			
3.1	Contrast-enhanced multiphasic cross-sectional imaging (CT or MRI) should be done before TIPS implantation	Low	Strong
3.2	The presence of portal cavernoma is technically challenging but not an absolute contraindication for TIPS implantation	High	Strong
(b) Cardiopulmonary assessment			
4.1	Comprehensive cardiac evaluation, including echocardiography, for any structural or functional cardiac abnormalities should be undertaken before elective TIPS implantation	Moderate	Strong
4.2	Detailed cardiac evaluation (cardiology consultation) should be undertaken before elective TIPS implantation if the patient has RVSP > 45 mmHg or TAPSE < 1.6 cm	Low	Strong
4.3	Salvage TIPS implantation in patients with acute variceal bleeding should not be delayed to await echocardiography	Low	Strong
4.4	Patients with mild pulmonary hypertension, moderate valvular disease or systolic or diastolic dysfunction before TIPS implantation should have echocardiographic assessment 3 months after TIPS implantation* ^*^ Mild pulmonary hypertension is defined by echocardiography as an estimated pulmonary artery systolic pressure (PASP) of 36–50 mmHg or a tricuspid regurgitant (TR) velocity of 2.9–3.4 m/s. Moderate valvular disease denotes valve lesions that are clinically significant but not severe and is defined by guideline-based quantitative thresholds (examples include aortic stenosis with valve area 1.0–1.5 cm^2^ or mean gradient 20–39 mmHg; mitral regurgitation with effective regurgitant orifice area 0.20–0.39 cm^2^ or regurgitant volume 30–59 mL/beat; and aortic regurgitation with vena contracta 0.3–0.6 cm or regurgitant volume 30–59 mL/beat). Systolic dysfunction is defined as left ventricular ejection fraction (LVEF) < 50% (mild 41–49%, moderate 30–40%, severe < 30%), while diastolic dysfunction is diagnosed when at least two standard echocardiographic parameters are abnormal (for example, average *E*/*e*′ > 14, septal *e*′ < 7 cm/s, lateral *e*′ < 10 cm/s, left atrial volume index > 34 mL/m^2^, or TR velocity > 2.8 m/s), with grading I–III according to accepted American Society of Echocardiography (ASE) and European Association of Cardiovascular Imaging (EACVI) criteria, which is endorsed by the Cardiac Society of Australia and New Zealand (CSANZ)	Low	Strong
(c) Nutritional assessment			
5.1	Malnutrition, sarcopenia and frailty should be assessed and managed in patients who are being considered for elective TIPS implantation	Low	Strong
(d) Renal function assessment			
6.1	Renal function should be evaluated in all patients before undergoing TIPS implantation	Low	Strong
6.2	For patients undergoing drainage of ascites during the TIPS procedure, albumin should be given according to current guidelines	High	Strong
(e) Hepatic encephalopathy			
7.1	Patients and their carers should be counseled that TIPS implantation is associated with a risk of hepatic encephalopathy (the incidence of overt hepatic encephalopathy is between 25 and 50%)	High	Strong
7.2	Baseline assessment for elective TIPS implantation should include assessment for covert and overt hepatic encephalopathy, ideally with at least two different modalities.* * Psychometric Hepatic Encephalopathy Score, Stroop testing, critical flicker frequency, and spectral-enhanced or quantitative electroencephalography	Moderate	Strong
7.3	For patients with a history of overt hepatic encephalopathy who are undergoing elective TIPS implantation, rifaximin 550 mg twice daily started within 14 days before TIPS placement and maintained for 6 months may reduce post-TIPS hepatic encephalopathy	High	Strong
7.4	There is no specific age cut-off that precludes TIPS implantation, even though increase in age is associated with increased risk of post-TIPS hepatic encephalopathy	Moderate	Strong
7.5	Embolisation of functionally significant spontaneous portosystemic shunts at the time of TIPS implantation may reduce the risk of post-TIPS hepatic encephalopathy	Moderate	Strong
**Contraindications**			
8.1	Moderate to severe pulmonary hypertension is a contraindication to TIPS implantation	Moderate	Strong
8.2	Advanced congestive heart failure (ACC/AHA stage C or D) is a contraindication to TIPS implantation	Moderate	Strong
8.3	Severe cardiac valvular insufficiency (ACC/AHA stage C or D) is a contraindication to TIPS implantation	Moderate	Strong
8.4	Severe or refractory overt hepatic encephalopathy (West Haven Criteria ≥ 2), except in the setting of acute variceal bleeding, is a contraindication to TIPS implantation	Moderate	Strong
8.5	Uncontrolled systemic infection or sepsis is a contraindication to TIPS implantation	Moderate	Strong
8.6	Anatomical preclusions to placement of the TIPS stent, including polycystic liver disease or extensive hepatic malignancy, are contraindications to TIPS implantation	Moderate	Strong
8.7	Unrelieved biliary obstruction is a relative contraindication to TIPS implantation	Moderate	Strong
8.8	Uncorrectable severe coagulopathy is a relative contraindication to TIPS implantation	Moderate	Strong
8.9	Elective TIPS implantation can be considered in patients presenting with acute variceal bleeding and renal dysfunction that is not classified as intrinsic renal disease (stage 4 or 5)	Low	Strong
**Consent**			
9.1	Additional or adjunctive procedures to be performed in conjunction with TIPS implantation, such as paracentesis or transjugular biopsy, should be included in the consent process	Low	Strong
9.2	Informed consent should follow established local and international principles and guidelines and be specific to the TIPS procedure	Moderate	Strong
**Best procedural practice**			
*Anesthesia and sedation*			
10.1	TIPS implantation should be performed either under general anesthesia or with deep sedation	Low	Strong
**Blood products**			
11.1	Viscoelastic testing should be considered to guide correction of coagulopathy	Moderate	Strong
11.2	Platelet transfusion may be considered if the patient’s platelet count is < 50 × 10^9^/L	Low	Weak
**Procedure**			
(a) Stents			
12.1	All elective TIPS procedures should be performed using ePTFE-lined stent grafts	High	Strong
12.2	For high-risk patients (with mild pulmonary hypertension or systolic or diastolic dysfunction), a stent of 8 mm diameter should be used to prevent worsening of cardiac function and reduce the risk of post-TIPS heart failure	Low	Strong
(b) Measurement of PSPG before and after TIPS			
13.1	PSPG should be measured both before and after TIPS placement	Moderate	Strong
13.2	In patients with acute variceal bleeding, the goal of TIPS is to achieve an absolute PSPG reduction to < 12 mmHg or a relative reduction in PSPG of > 50% from the pre-TIPS baseline gradient	High	Strong
13.3	For TIPS indications other than variceal bleeding, reductions in PSPG must be individualized and need to balance efficacy and the risk of postoperative hepatic encephalopathy and deterioration in liver function	Low	Strong
**Postoperative care and follow-up**			
*Post-TIPS monitoring*			
14.1	After TIPS implantation, patients should be followed up by a gastroenterologist or hepatologist and an interventional radiologist to monitor for ongoing procedural complications and liver disease	Low	Strong
14.2	After TIPS implantation, the patient’s kidney function should be closely monitored for contrast-related injury or AKI	Moderate	Strong
14.3	Post-TIPS hepatic encephalopathy that is resistant to medical therapy warrants consideration of embolisation of spontaneous portosystemic shunts or endovascular stent reduction or closure	Low	Strong
**Doppler ultrasound**			
15.1	Doppler ultrasound surveillance should be done 1 week after the TIPS implantation, then at 3 months and 6 months, and thereafter at 6-monthly intervals. Each ultrasound study should measure and record shunt patency and velocity	Low	Strong
15.2	Doppler ultrasound findings suggesting TIPS dysfunction include alteration of intrahepatic portal vein flow direction and abnormal portal vein velocity. These should both be measured and documented at every surveillance Doppler ultrasound study. Abnormal flow direction or velocity should prompt review at a multidisciplinary meeting	Low	Strong
**Venography**			
16.1	Routine venography is not necessary after TIPS implantation, except for patients with prothrombotic conditions, such as Budd–Chiari syndrome (BCS) or splanchnic vein thrombosis	Low	Strong
16.2	Doppler ultrasound findings suggestive of TIPS dysfunction, TIPS stenosis or recurrence and persistence of complications of portal hypertension should trigger investigation with TIPS venography, manometry and PSPG remeasurement, and revision as appropriate	Low	Strong
**Indications for TIPS**			
*TIPS for portal hypertensive bleeding*			
(A) Acute variceal bleeding			
17.1	Patients with acute variceal bleeding who achieve endoscopic hemostasis but are at high risk of rebleeding* should undergo pre-emptive TIPS implantation * Those with Child–Pugh class C cirrhosis (score of 10–13) or Child–Pugh class B cirrhosis (score of 8–9) with active bleeding, and/or a MELD score ≥ 19 at the time of initial endoscopy	High	Strong
17.2	Salvage TIPS implantation should be performed urgently for suitable patients who have variceal bleeding refractory to standard care	High	Strong
17.3	TIPS implantation should be performed for suitable patients with recurrent variceal bleeding despite medical and endoscopic therapy	High	Strong
17.4	Salvage TIPS implantation should not be performed in a patient with a Child–Pugh score > 13, a MELD score > 30 and a lactate level > 12 mmol/L, unless liver transplantation is planned in the short term	Moderate	Strong
17.5	Concurrent variceal embolisation can be considered in patients undergoing TIPS implantation for management of variceal bleeding	High	Strong
(B) Secondary prevention of variceal bleeding			
18.1	For secondary prevention of gastric variceal bleeding, TIPS implantation can be considered for patients with recurrent bleeding despite endoscopic injection therapy or for selected patients with large or multiple gastric varices	Moderate	Strong
(C) Gastric varices			
19.1	TIPS implantation should not be used for primary prophylaxis of gastric variceal bleeding	Moderate	Strong
19.2	TIPS implantation can be considered for treatment of gastric variceal bleeding if this cannot be managed endoscopically	Moderate	Strong
19.3	TIPS implantation is the preferred treatment option over spontaneous shunt obliteration for patients with profound portal hypertension complications, such as large-volume ascites or significant esophageal varices, and for patients with PVT	Low	Strong
(D) Ectopic varices and portal hypertensive gastropathy			
20.1	Patients with suspected bleeding from ectopic varices (including intestinal, stomal and anorectal varices) should be reviewed by a multidisciplinary team, with consideration given to either endoscopic or endovascular treatment and an individualized treatment plan, before consideration of TIPS implantation	Low	Strong
20.2	TIPS implantation can be considered for patients with bleeding from portal hypertensive gastropathy that is refractory to medical therapy	Low	Strong
**TIPS for ascites or hepatic hydrothorax**			
21.1	TIPS implantation can be used for selected patients with cirrhosis and refractory ascites	Moderate	Strong
21.2	For patients with ascites or hepatic hydrothorax, sodium restriction and diuretics may still be required after TIPS implantation	Low	Strong
21.3	Patients with refractory hepatic hydrothorax should be considered for TIPS implantation	High	Strong
21.4	For patients with cirrhosis and diuretic-resistant ascites or hepatic hydrothorax who are undergoing elective TIPS implantation, stepwise stent dilatation should be performed	High	Strong
**TIPS for hepatorenal syndrome**			
22.1	HRS is not an absolute contraindication for TIPS implantation in patients with indications such as variceal hemorrhage or refractory ascites	High	Strong
22.2	There is insufficient evidence to recommend TIPS implantation for patients with HRS–AKI	Low	Weak
**TIPS for hepatopulmonary syndrome**			
23.1	There is insufficient evidence to recommend TIPS implantation for patients with hepatopulmonary syndrome	Low	Strong
**Other indications for TIPS**			
(i) TIPS for Budd–Chiari syndrome			
24.1	Patients with BCS undergoing elective TIPS implantation should be managed in experienced centers that perform complex TIPS procedures. Ideally, such centers would have a liver transplant unit or be linked to a transplant service	Low	Strong
(ii) TIPS for portal vein thrombosis			
25.1	Portal vein recanalisation and TIPS implantation can be considered for patients with complete PVT	High	Strong
25.2	TIPS has an emerging role in the management of patients with complicated portal-mesenteric thrombosis	Moderate	Strong
(iii) TIPS for idiopathic non-cirrhotic portal hypertension			
26.1	Among patients with idiopathic non-cirrhotic portal hypertension, TIPS implantation can be considered for the same indications as for patients with cirrhotic portal hypertension	Low	Strong
**Prophylactic TIPS for non-hepatic surgery**			
27.1	The role of TIPS implantation in reducing portal pressure before non-hepatic abdominal surgery is not clearly defined	Low	Strong

In accordance with GRADE guidance, strong recommendations based on low-certainty evidence were permitted when one or more of the following conditions were met: (i) the balance of benefits and harms was judged to be clear and consistent across available evidence; (ii) the intervention addressed outcomes of critical clinical importance, including morbidity, mortality, or irreversible harm; (iii) there was substantial concordance between indirect evidence, pathophysiological rationale, and clinical experience; (iv) patient values and preferences were considered unlikely to vary meaningfully; and/or (v) failure to act was judged to pose a greater risk than implementation of the recommendation. In such cases, low certainty of evidence reflected limitations in study design, imprecision, or indirectness rather than uncertainty regarding the direction of effect. The rationale for issuing a strong recommendation in the context of low-certainty evidence was explicitly discussed by the panel and documented during the consensus process.

**Fig. 2:**
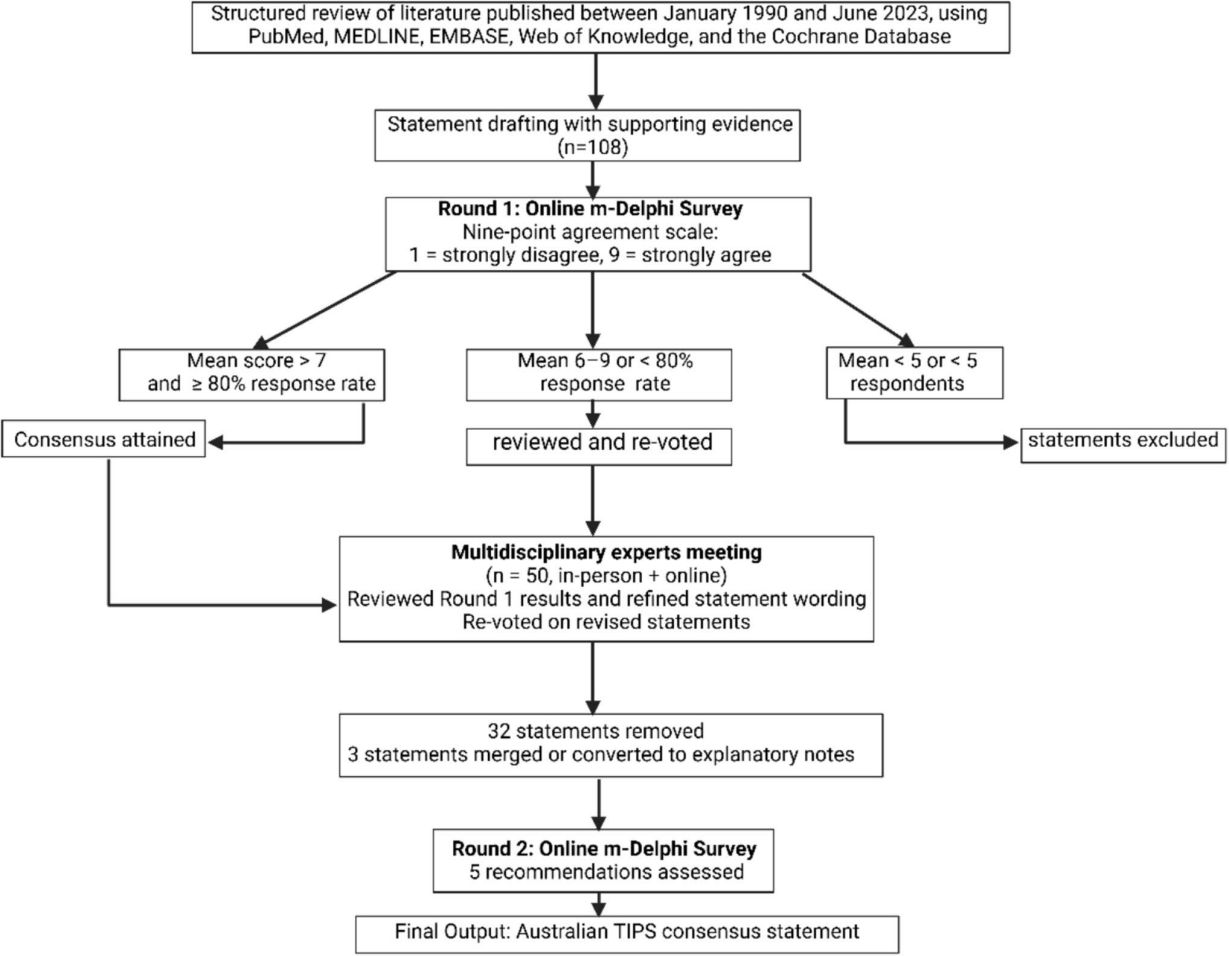
Overview of the m-Delphi consensus process for the Australian TIPS consensus statement

Statements were categorized into four overall clinical domains related to TIPS, as follows: (1) preparation for TIPS, patients’ selection, and pre-TIPS workup, (2) best procedural practice, (3) postoperative care and follow-up, and (4) indications for TIPS. Recommendation statements were tailored to the Australian context where relevant. An 80% consensus was required for statements to progress to the modified Delphi (*m-*Delphi) consensus rounds. Participants were asked in *m*-Delphi round 1 to rate their agreement with each statement based on a 9-point scale, with 1 being “strongly disagree” and 9 being “strongly agree”. Consensus was defined as a mean score above 7 (indicating agreement) with responses from at least 80% of participants. Statements scoring between 6 and 9, or those not meeting the 80% response criterion, were subsequently reviewed and subjected to formal voting during a hybrid online and in-person meeting. Recommendations with a mean score < 5 with/without disagreement of participants or if the number of responders is less than 5 has not automatically progressed to hybrid online–face-to face meeting (Fig. 2). All participants completed a standardized conflict-of-interest disclosure form prior to participation. Disclosures were prospectively documented in the project record. Members were permitted to abstain from voting on any statement for which they considered themselves to lack sufficient expertise. Dissenting comments and reasons for disagreement were reviewed collectively by the panel.

Statements that did not meet the predefined consensus threshold were revised based on substantive feedback and recirculated for re-rating in subsequent m-Delphi rounds.

GESA convened multidisciplinary expert groups to discuss several identified topics at a hybrid online and face-to-face meeting held in Sydney on 24 May 2024. At this meeting, the preliminary ratings from the first *m*-Delphi round were discussed, statements with suboptimal wording were refined, and a second round of formal voting was held to re-rate statements through equally weighted voting. Participants (*n* = 50) who could not attend in person were given the option to attend online via videoconferencing for real-time discussion on each proposed statement. The complete list of participants—including names, specialties, institutions, and states—is provided in Supplementary Table 1 (Acknowledgement of participation). A second online questionnaire (*m*-Delphi round 2) followed the hybrid meeting.

Results from both *m*-Delphi rounds have provided a practical approach and best practice recommendations for the clinical assessment and management of patients undergoing consideration for the TIPS procedure. Summary of recommendations can be found in Table 1.

Following the modified Delphi process, the final recommendations underwent independent external review by international experts to confirm validity, clarity, and applicability before publication.

## Preparation for TIPS, patient selection and pre-TIPS workup

### Preparation for TIPS

A multidisciplinary and team-based approach to TIPS is of crucial throughout all stages of TIPS planning, management, and evaluation [5, 16]. The decision to implant a TIPS (whether emergent or non-emergent) should initially involve multidisciplinary team discussions with, as a minimum, one hepatologist and one interventional radiologist who have proven procedural expertise. Where appropriate, and based on individual factors, the multidisciplinary team discussions should also involve various other specialists, such as a liver transplant surgeon, liver transplant physician, cardiologist, anesthesiologist, intensivist, hematologist or nephrologist. Longitudinal care for patients who undergo TIPS involves a range of specialists and allied health professionals, to minimize the risk of complications and optimize patient outcomes.

TIPS implantation demands a high degree of technical skill and clinical proficiency to achieve optimal patient outcomes. It should therefore be undertaken by appropriately trained and experienced proceduralists at centers with relevant experience in performing the procedure, and where specialist services for managing complications are available [17, 18]. Procedural and post-procedural complications of TIPS implantation are listed in Table 2.

**Table 2:** Complications of the transjugular intrahepatic portosystemic shunt (TIPS) procedure

Complications	Classification
• Fever • Hemobilia • Hepatic encephalopathy responsive to medical therapy • Transient pulmonary edema • Entry-site hematoma	Minor
• Hemoperitoneum • Biliary peritonitis • Hepatic ischemia or infarction • Gall bladder puncture • Hepatic artery branch trauma • Hepatic vein thrombosis • Capsule transgression • Portal vein injury • Non-target shunt insertion (mispositioning) • Allergic anaphylactic reaction to medications or contrast dye	Major—procedural
• Hepatic encephalopathy unresponsive to standard medical therapy • Venous biliary fistulae • Shunt migration • Renal failure (contrast-induced) • Liver failure • Heart failure • TIPS occlusion • TIPS infection (endotipsitis)	Major—post-procedural
• Death	Major—procedural and post-procedural

### Patient selection

Patient selection for TIPS is a multidisciplinary decision that involves demographic, clinical and laboratory parameters and preoperative considerations, as well as standard scoring systems for patients with cirrhosis. These include the model for end-stage liver disease (MELD), Child–Pugh score, chronic Liver failure consortium acute-on-chronic liver failure (CLIF-C ACLF) score, modified TIPS score (MOTS), Emory model, Bonn TIPS early mortality (BOTEM) score, platelet–albumin–bilirubin index (PALBI) and Freiburg index of post-TIPS survival (FIPS) [19–25]. Recently, the Elderly Patients Calculator TIPS (ExPeCT) model was developed for mortality prediction in patients aged ≥ 70 years with an indication for TIPS [26].

Prognostic scoring to assess candidacy for TIPS should be individualized to the patient’s clinical context and interpreted within a multidisciplinary decision-making framework. Historically, MELD score has been the strongest predictor of 90-day mortality after TIPS. Some studies suggest that TIPS should not be undertaken when the MELD score is ≥ 18 for patients with ascites [27]. However, the role of MELD score in patient candidacy for TIPS implantation remains unclear. Recently, the FIPS score has demonstrated superiority to established scoring systems (Child–Pugh, MELD and MELD-Sodium scores and the bilirubin–platelet model) for identifying high-risk patients with a worse prognosis after elective TIPS implantation for variceal bleeding or refractory ascites [25, 28, 29]. Nonetheless, the FIPS score demonstrates limited prognostic discrimination in patients undergoing pre-emptive TIPS implantation. A study in a Chinese cohort of 536 patients undergoing TIPS found that risk stratification using the Child–Pugh score appeared more favorable than using the FIPS score [30]. Therefore, using prognostic scoring to assess candidacy for TIPS implantation can only guide clinical decision-making but the potential risks and benefits should be carefully evaluated for each individual patient. The prognostic scoring systems available for predicting survival after TIPS implantation are listed in Supplementary Table 2.

### Pre-TIPS workup

#### Imaging

Contrast-enhanced multiphasic cross-sectional (CT/MRI) series and Doppler ultrasound examinations should include portal venous phase imaging to adequately define portal veins, hepatic veins, and the parenchyma of the liver and review any vascular and parenchymal anatomic variations to determine suitability of TIPS implantation. For patients who require emergency TIPS implantation, bedside echocardiography of the heart and Doppler ultrasound of the liver are a possible alternative [31, 32].

#### Cardiopulmonary assessments

The complex hemodynamic changes observed in cirrhosis and portal hypertension can result in impaired cardiac function, a syndrome known as cirrhotic cardiomyopathy. This includes a cluster of cardiovascular abnormalities, such as impaired cardiac contractility with systolic and/or diastolic dysfunction, as well as electromechanical abnormalities (i.e., prolonged QT interval) in the absence of other known causes of cardiac disease [33–35]. Cirrhotic cardiomyopathy complicates several therapies used for treating patients with cirrhosis and confers an increased risk of post-TIPS heart failure. Diastolic dysfunction has been identified as predictor of cardiac events post-TIPS implementation [36, 37]. Therefore, cardiovascular assessment is essential before TIPS implantation, as increased portosystemic shunting is associated with worsening of hyperdynamic syndrome, as seen by a marked rise in preload and cardiac output, combined with a concomitant decline in systemic vascular resistance [37].

Cardiac decompensation occurs in up to 20% of patients within the first year after TIPS implantation [38]. Right-sided heart failure may be triggered not only in patients with pre-existing overt heart failure or severe tricuspid regurgitation, but also in those with subclinical cardiac dysfunction or moderate to severe portopulmonary hypertension [39–41]. Hence, a comprehensive cardiac history should be taken, and investigations to evaluate any structural or functional cardiac abnormalities should be done for all patients undergoing elective TIPS implantation. Investigations should incorporate 12-lead electrocardiography, measurement of serum N-terminal pro-B-type natriuretic peptide level and echocardiographic measurement of both left and right ventricular function. Transthoracic echocardiographic assessment should include assessment of left ventricular ejection fraction and measurement of left ventricular diastolic function, left ventricular global longitudinal strain, tricuspid annular plane systolic excursion (TAPSE) and right ventricular systolic pressure (RVSP) [38, 42, 43]. Bedside echocardiography and Doppler ultrasound of the liver may be indicated for patients with acute variceal bleeding who are undergoing emergency TIPS, but these investigations should not preclude or delay an emergency TIPS procedure.

#### Nutritional assessment

Sarcopenia, frailty, and malnutrition are prevalent among patients with decompensated cirrhosis [44]. Muscle wasting is associated with an increased risk for the development of HE after TIPS implantation [45, 46]. Recent international guidelines recommend that skeletal mass index be quantified by CT imaging given that this method is the most consistent and reproducible. A detailed dietic assessment can be preserved for patients at risk of malnutrition [4]. This is even more relevant for patients with ascites, as underlying malnutrition is likely to contribute to its severity. Alcohol relapse is common after TIPS implantation, underscoring the need for careful risk assessment, especially in patients scheduled for elective procedures [47].

#### Renal function assessment

Physiologically, TIPS implantation may halt the decline of renal function and lead to improvement, owing to decreased effective circulating volume [48–55].

Renal function should be evaluated before TIPS, either through measurement of serum creatinine level or using glomerular filtration rate (estimated or measured), as contrast-induced injury and acute kidney injury (AKI) can occur after TIPS implantation [56].

To date, most RCTs of TIPS have excluded patients with advanced renal disease [57–70]. However, several studies have reported that the incidence of post-TIPS AKI was about 17% [56, 71–73]. This proportion does not take into consideration whether these represent true cases of AKI occurring after TIPS, a result of pre-TIPS intrinsic kidney disease or an AKI from pre-existing stable chronic renal disease.

The current opinion of both the ALTA Consortium and GESA is that there is no absolute serum creatinine cut-off level, chronic kidney disease stage or factors such as presence or absence of renal replacement therapy that contraindicate TIPS implantation. However, given that severe hepatic encephalopathy is common in the presence of severe (stage 4 or 5) intrinsic renal disease [74, 75], we recommend against TIPS implantation in this group of patients. Mitigating post-TIPS AKI and risk of progression to acute or chronic kidney disease is crucial. For patients undergoing TIPS implantation for ascites, human albumin infusions can be considered for those having large-volume (usually > 5 L) paracentesis, to reduce the risk of paracentesis-induced circulatory dysfunction and AKI. Infusions may occur 24 h before, or during, TIPS implantation [76–79].

#### Hepatic encephalopathy

Hepatic encephalopathy is a frequent complication of all portosystemic shunts, including TIPS [80]. An episode of overt hepatic encephalopathy occurs in up to 50% of patients post-TIPS implantation [64, 81–90].

Numerous patient factors contribute to the development of post-TIPS hepatic encephalopathy, including older age, severity of liver disease (Child–Pugh class C, Child–Pugh score ≥ 10 or MELD score > 18), prior hepatic encephalopathy, shunt size, sarcopenia, elevated creatinine level, hyponatremia and low portosystemic pressure gradient (PSPG) after TIPS placement [45, 75, 90]. Therefore, patients scheduled for non-urgent TIPS implantation should be routinely assessed and screened for the presence or a history of overt or covert (subclinical) hepatic encephalopathy [91, 92].

Typically, hepatic encephalopathy presents episodically or continuously with detectable neuropsychiatric abnormalities. In contrast, minimal hepatic encephalopathy is often overlooked in practice and can only be diagnosed with neuropsychometric testing. Screening should ideally involve at least two of: the Psychometric Hepatic Encephalopathy Score, Stroop testing, critical flicker frequency, and spectral-enhanced or quantitative electroencephalography. Covert hepatic encephalopathy carries an increased risk of falls, driving impairment and diminished quality of life [93]. The diagnosis of covert hepatic encephalopathy in the absence of acute decompensation—a relative contraindication to elective TIPS—may guide discussions with patients, caregivers and clinical teams regarding the utility of TIPS [84, 94].

TIPS implantation is among the most technically delicate interventional radiological procedures. The initial stent diameter is a key determinant in reducing the postoperative risk of hepatic encephalopathy. Controlled-expansion stents incremental and reliable adjustment of the stent diameter. Small portosystemic shunts are associated with a lower incidence of hepatic encephalopathy, though this may compromise the degree of portal pressure reduction and decompression [95–97]. For patients with a history of overt hepatic encephalopathy, receiving rifaximin before TIPS implantation may significantly reduce the risk of hepatic encephalopathy after the procedure [92].

Despite careful patient selection and investigation for hepatic encephalopathy before TIPS implantation, the onset of hepatic encephalopathy after TIPS is common, although it is usually short-lived. Basic hepatic encephalopathy management should include correction of underlying biochemical abnormalities, cessation of night sedation and prescription of lactulose at the first episode of overt hepatic encephalopathy. Proton pump inhibitors, if prescribed, should be ceased. For subsequent episodes of overt hepatic encephalopathy, both rifaximin and lactulose are recommended [91]. If hepatic encephalopathy remains refractory to medical therapy, treatment should include endoscopy for surveillance and treatment of high-risk varices before undertaking collateral large (> 6 mm) spontaneous portosystemic shunt embolisation, followed by TIPS stent reduction (recalibration) or occlusion [75, 98]. Supplementary Table 3 summarizes recent studies on HE incidences and outcomes post-TIPS implantation.

#### Contraindications

Contraindications for TIPS can be found in Table 3.

**Table 3:** Contraindications for TIPS

• Moderate to severe pulmonary hypertension is a contraindication to TIPS implantation
• Advanced congestive heart failure (ACC/AHA stage C or D) is a contraindication to TIPS implantation
• Severe cardiac valvular insufficiency (ACC/AHA stage C or D) is a contraindication to TIPS implantation
• Severe or refractory overt hepatic encephalopathy (West Haven Criteria ≥ 2), except in the setting of acute variceal bleeding, is a contraindication to TIPS implantation
• Uncontrolled systemic infection or sepsis is a contraindication to TIPS implantation
• Anatomical preclusions to placement of the TIPS stent, including polycystic liver disease or extensive hepatic malignancy, are contraindications to TIPS implantation
• Unrelieved biliary obstruction is a relative contraindication to TIPS implantation
• Uncorrectable severe coagulopathy is a relative contraindication to TIPS implantation

#### Consent

Informed consent is an essential component of patient care, including before a TIPS procedure. In accordance with local and international guidelines, health care providers must ensure that patients fully understand the risks and benefits of the TIPS procedure, as well as any available alternative treatments [4, 16, 99–105]. Patients should also be made aware of potential side effects of TIPS implantation, including hepatic encephalopathy, to ensure they can make a well-informed decision about their treatment [4].

A fact sheet that can be provided to patients to help them understand the TIPS procedure and give their informed consent is available at: https://www.gesa.org.au/public/13/files/Education%20%26%20Resources/Patient%20Resources/TIPS/GESA%20TIPS%20Patient%20Resource.pdf.

## Section 2 Best procedural practice

### Anesthesia and sedation

TIPS implantation should be performed either under general anesthesia or with deep sedation.

### Blood products

Evidence regarding management of procedural bleeding risk in patients undergoing the TIPS procedure is limited, particularly for correction of thrombocytopenia, but there is some evidence that viscoelastic testing can guide correction of coagulopathy [106–111].

### Procedure

#### Stents

While bare-metal stents were previously standard, expanded polytetrafluoroethylene (ePTFE)-covered stents have become the gold standard in routine practice, due to superior patency, better ascites control, reduced risk of rebleeding, and overall cost-effectiveness [88, 112–122]. We recommend using stents with an 8 mm diameter in patients with high cardiac risk (mild portopulmonary hypertension or systolic or diastolic dysfunction) to prevent worsening of cardiac function and reduce the risk of post-TIPS heart failure [123, 124].

#### Measurement of PSPG before and after TIPS

Post-TIPS PSPG is important for predicting rebleeding risk and postoperative complications, such as hepatic encephalopathy and deterioration in hepatic function. One study identified that a PSPG > 12 mmHg after TIPS implantation (> 24 h of follow-up) was associated with an 8.5-times higher risk of recurrence of portal hypertension complications (variceal bleeding or ascites) [125]. PSPG should be measured before and after TIPS implantation, with the aim of achieving an absolute reduction in PSPG to < 12 mmHg, or a relative reduction of > 50% from the pre-TIPS baseline, in patients with acute variceal bleeding [39, 125].

PSPG should be measured as the difference in pressure between the main portal vein and the confluence of the hepatic vein and inferior vena cava (IVC). If measurement of IVC pressure is not feasible, right atrial pressure can be used instead; however, this may lead to an overestimation of PSPG [31, 39, 126].

## Section 3 Postoperative care and follow-up

### Post-TIPS monitoring

Patients post-TIPS should be followed with gastroenterologist/hepatologists and interventional radiologist to monitor for ongoing procedural complications as well as ongoing liver disease. Kidney function should be closely monitored in patients post-TIPS for contrast-related injury, or AKI.

### Doppler ultrasound

In the era of ePTFE-covered stents, early TIPS dysfunction due to thrombosis rarely occurs. Hence, early Doppler ultrasound within about 48 h is of limited utility soon after TIPS insertion. Proactive follow-up with Doppler ultrasound typically takes place 1 week after TIPS placement, then routinely at 3 months, 6 months and 6-monthly intervals thereafter [127]. Early ultrasound findings suggesting TIPS dysfunction should prompt endovascular evaluation especially when combined with recurrent of portal hypertension-related symptoms (e.g., ascites and variceal bleeding) [128]. Doppler ultrasound findings suggesting of TIPS dysfunction includes alteration of intrahepatic portal vein flow direction or abnormal velocity within TIPS (A mean maximum flow velocity (mVPmax) at the portal vein < 28 cm/s when flow is hepatofugal or mVPmax < 39 cm/s when flow is hepatopetal).

### Venography

Indications for venography include abnormal Doppler parameters, such as markedly elevated or diminished velocities within the shunt, reversed portal venous flow or the recurrence of portal hypertensive complications, including refractory ascites or variceal hemorrhage. Catheter-based venography, typically performed in conjunction with PSPG assessment, constitutes the reference standard for evaluating TIPS patency and hemodynamic function. This technique permits direct visualization of luminal stenosis, thrombosis, or structural anomalies within the shunt and facilitates immediate endovascular intervention when indicated (e.g., balloon angioplasty or secondary stent placement) if indicated [129–133].

## Section 4 Indications

### TIPS for portal hypertensive bleeding

#### Acute variceal bleeding and TIPS

Acute variceal bleeding is a life-threatening complication of cirrhosis. Its annual incidence is 10–15%, and 6-week mortality is about 15–20% [134]. A landmark 1981 study showed that mortality in patients with acute variceal bleeding is concentrated within the first 3 days [135]. At present, the standard of care for patients with acute variceal bleeding includes a combination of restrictive transfusion strategy (packed red blood cells, with a target hemoglobin level of 70–80 g/L), endoscopic variceal ligation, antibiotic prophylaxis and vasoactive drugs (e.g., terlipressin, somatostatin or octreotide), and salvage balloon tamponade or insertion of self-expanding metal stents [136]. Nonetheless, initial endoscopic and medical treatment fails in 10–20% of patients, which is associated with a high short-term risk of further liver decompensation and death. Most patients with acute variceal bleeding have advanced (Child–Pugh class C) cirrhosis, and rescue TIPS implantation results in high mortality in these patients. Numerous endovascular obliteration or embolisation techniques, such as retrograde transvenous obliteration (plug-assisted, coil-assisted or balloon) or antegrade transvenous obliteration, have been used as alternative or complementary treatments to TIPS for managing variceal bleeding. High MELD scores and lactate levels have been associated with mortality in patients with cirrhosis treated with salvage TIPS implantation [137] (Table 4).

**Table 4:** Summary of clinical indications, evidence, and expected outcomes for TIPS

Indication	Evidence and expected outcomes
Acute variceal bleeding—pre-emptive TIPS	RCTs and meta-analyses show reduced rebleeding and improved short-term survival in high-risk patients (Child–Pugh C 10–13 or B 8–9 with active bleeding). Some trials report increased HE; HVPG > 20 mmHg predicts benefit, but HVPG not widely available
Acute variceal bleeding—salvage (rescue) TIPS	Used when standard medical/endoscopic therapy fails. Controls bleeding effectively, but high mortality in advanced liver disease (high MELD, lactate). Alternative bridging techniques include balloon tamponade or stents
Secondary prevention of variceal bleeding	Two RCTs support TIPS to reduce rebleeding; increased HE risk; survival/QoL impact inconsistent. Recommended as second-line therapy after failure of endoscopic and pharmacologic therapy
Gastric varices (GOV2/IGV1)	Small RCTs and observational studies. Pre-emptive TIPS with glue ± NSBB improve rebleeding-free survival; higher HE risks than BRTO; survival similar. TIPS preferred in severe portal hypertension; BRTO for patients with prior HE
Ectopic varices/portal hypertensive gastropathy	Case reports/small series. TIPS can achieve hemostasis, but evidence limited; consider when local therapy fails
Refractory/recurrent ascites	Meta-analyses show improved ascites control vs LVP; increased HE risk; transplant-free survival benefit variable. Typical indication: ≥ 3 LVP in 1 year; hepatic hydrothorax evidence limited
Hepatorenal syndrome (HRS-AKI)	Meta-analysis shows renal function may improve post-TIPS, insufficient evidence for routine use. Consider experimental/selected patients only
Hepatopulmonary syndrome (HPS)	Insufficient evidence: TIPS not routinely recommended
Budd–Chiari syndrome (BCS)	Case series show good long-term outcomes; 5-year survival > 70%; re-intervention often needed. TIPS (or DIPS if hepatic veins occluded) indicated if medical/revascularization therapy fails
Portal vein thrombosis (PVT)	Feasible and may reduce portal pressure; evidence limited. Consider in extensive acute/chronic PVT or bowel ischemia when anticoagulation insufficient
Idiopathic non-cirrhotic portal hypertension (INCPH)	Observational studies only; limited evidence; use in selected patients
Prophylactic TIPS (peri-operative/non-hepatic surgery)	Very limited data; insufficient evidence to recommend routine prophylactic use

In 2004, the first study on pre-emptive TIPS implantation for acute variceal bleeding was published [61]. In this RCT, TIPS implantation was performed for acute variceal bleeding within 72 h of diagnostic endoscopy in 26 hemodynamically stable patients, with a hepatic venous pressure gradient > 20 mmHg, who had received standard care and were likely to be at high risk of future rebleeding and bleeding-related mortality. In these patients, TIPS placement within 24 h of admission led to a significant reduction in mortality and rebleeding, compared with standard-of-care measures. However, given that routine hepatic venous pressure gradient measurement is not readily available at most institutions, pre-emptive TIPS implantation was not championed, despite this landmark trial.

In 2010, a second RCT showed that pre-emptive TIPS implantation within 72 h of initial upper endoscopy in 63 patients admitted with acute variceal bleeding and Child–Pugh class B cirrhosis (with active bleeding at endoscopy) or class C cirrhosis (regardless of bleeding status at time of endoscopy) was associated with significant reductions in rebleeding and failure to control bleeding, and improved survival, with no increased risk of hepatic encephalopathy [85]. These results were later confirmed in a larger trial of 132 patients [86].

Further, individual patient data meta-analysis of three RCTs and five observational studies, involving 1327 patients with cirrhosis (of whom 310 received pre-emptive TIPS implantation and 1017 received vasoactive drugs and endoscopy), concluded that patients with Child–Pugh class C cirrhosis (score 10–13) or class B (score 8–9) presenting with active variceal bleeding at endoscopy are at high risk of rebleeding and are the most likely to benefit from pre-emptive TIPS implantation [138]. Patients who do not fulfill these criteria should be evaluated for rescue TIPS [139]. Independent of Child–Pugh class, any patient presenting with uncontrolled acute variceal bleeding at endoscopy warrants referral for salvage TIPS. However, a more recent two-center open-label RCT of 58 patients found that pre-emptive TIPS reduced variceal bleeding but had no effect on survival and, conversely, increased the risk of hepatic encephalopathy [140].

A growing body of observational studies suggests that the presence of hyperbilirubinemia, hepatic encephalopathy or acute-on-chronic liver failure at time of bleeding has no impact on patients’ survival when they are managed with pre-emptive TIPS implantation [1, 141, 142].

Aside from the known role of TIPS in treating variceal bleeding, a recent meta-analysis of individual patient data, including 12 controlled studies involving 2338 patients, found that TIPS implantation for prevention of variceal rebleeding and refractory ascites can reduce the overall risk of further decompensation and improve survival, compared with standard care [143].

#### Secondary prevention of variceal bleeding

Guidelines from prominent international liver disease societies (AASLD, EASL and ALTA) consider TIPS implantation as second-line therapy for prevention of recurrent bleeding. This is based on two RCTs demonstrating the clinical utility of TIPS in reducing variceal rebleeding risk, with an increased risk of hepatic encephalopathy and no impact on survival or quality of life [144, 145].

#### Gastric varices

Limited evidence supports the effectiveness of TIPS or BRTO (balloon-retrograde transverse obliteration) for management of GVs. Available literature has shown that TIPS when compared with BRTO is associated with higher postoperative procedure-related complications, higher rebleeding risk, postoperative HE and hepatic ischemia without any difference in patient survival [146–153].

Pre-emptive TIPS (when combined with endoscopic cyanoacrylate injection (glue) with or without NSBBs) can be used as first-line therapy for GOV2 and/or IGV1 management. A recent RCT of small sample size (*n* = 21) demonstrated that the use of pre-emptive TIPS (within first 5 days of bleeding) with combined endoscopic (injection of glue) and pharmacological therapy (first, somatostatin or terlipressin; carvedilol after discharge (*n* = 11) is associated with a significantly higher rebleeding-free survival in patients with gastric fundal varices (IGV1/GOV 2), when compared with standard care (*n* = 10) in patients of Child–Pugh B and C scores [154]. Importantly, in this RCT, patients had bare stents that carry much poorer outcomes.

The role of TIPS for secondary prevention of GVs hemorrhages is not yet fully clear. The evidence supporting this approach is comprised of one RCT and two observational studies [155, 156]. The RCT has valuated TIPS, performed using legacy bare-metal stents in 35 patients, versus repeated endoscopic cyanoacrylate injection in 37 patients for the prevention of gastrofundal variceal rebleeding [157]. All patients initially achieved hemostasis with cyanoacrylate injection. The study demonstrated that TIPS was associated with a lower rate of variceal rebleeding but a higher incidence of hepatic encephalopathy, with no significant difference in overall survival between the two groups.

TIPS and BRTO are superior to endoscopic cyanoacrylate injection for secondary prevention of GV hemorrhage, with similar hemostasis rates and comparable complications. TIPS is preferred for patients with severe portal hypertension complications (e.g., large-volume ascites, significant EV, and PVT), while BRTO is better for those with a history of HE.

#### Ectopic varices, portal hypertensive gastropathy

The evidence for TIPS in management of ectopic varices is based on individual case reports or small retrospective case series.

### TIPS in ascites and hepatic hydrothorax

TIPS can be considered an effective alternative in patients who require large-volume paracentesis at frequent intervals. In contrast to variceal bleeding, there is lack of consensus on the optimum target PSPG when placing TIPS for refractory ascites. Several meta-analyses have demonstrated that TIPS led to better ascites control as compared to LVP but at the cost of increased risk of encephalopathy. Demonstrating an improvement in transplant-free survival has been by far more controversial, with some but not all RCTs demonstrating a benefit of TIPS implantation [57, 60, 65, 158]. A recent study by Bureau et al. assessed the outcomes of PTFE-covered TIPS versus large-volume paracentesis (LVP) with albumin infusions in patients with recurrent ascites. The study found that TIPS significantly improved 1-year transplant-free survival (HR 2.1; 95% CI 1.1–4.0) without a concomitant increase in the incidence of post-procedural HE [64].

With respect to recurrent ascites (≥ 3 large-volume paracenteses within 1 year) and TIPS, numerous meta-analyses have highlighted the key role of TIPS can play in prevention of recurrent ascites when compared to serial LVP.

Evidence supporting TIPS in the treatment of hepatic hydrothorax is limited to a few case reports and case series.

### TIPS for hepatorenal syndrome

Unlike pre-renal AKI, which results from hypoperfusion of the kidneys due to fluid or blood loss, HRS-AKI arises from a severe reduction in effective circulating volume secondary to systemic circulatory dysfunction.

Although renal function tends to improve post-TIPS—as demonstrated in a meta-analysis of nine studies, there is insufficient evidence to recommend TIPS in patients with HRS-AKI [159]. The use of TIPS in HRS remains experimental.

### TIPS for hepatopulmonary syndrome

There is insufficient evidence to recommend TIPS for hepatopulmonary syndrome [160–162].

### Other indications for TIPS

#### TIPS for Budd–Chiari syndrome

Patients who fail to respond to initial medical management and hepatic interventions, or who are not suitable candidates for percutaneous hepatic venous outflow revascularization, may benefit from TIPS to decompress congested hepatic segments. Due to the rarity of BCS, RCTs evaluating the use of TIPS for BCS do not exist. Large series of studies demonstrate that TIPS creation is associated with good overall long-term outcomes and 5-year survival rate exceeding 70%. Patients with BCS, however, require regular re-intervention to restore TIPS patency. A direct intrahepatic porto-caval shunts (DIPS) procedure, also known as transjugular transcaval intrahepatic portosystemic shunt (TTIPS) can be performed in patients with occlusion of all hepatic veins. Outcome in-patient with BCS who had TIPS placement is generally assessed by BCS-TIPS prognostic score [163].

#### TIPS for portal vein thrombosis

TIPS placement is technically feasible and may confer the additional benefit of controlling portal pressure in patients with acute and chronic PVT, especially patients with extensive PVT and bowel ischemia. However, the evidence to recommend TIPS remains low given the absence of prospective studies and a paucity of real-world data evaluating TIPS versus anticoagulation monotherapy.

#### TIPS and idiopathic non-cirrhotic portal hypertension

Evidence on utility of TIPS in INCPH is limited to few observational studies [164–169].

### Prophylactic TIPS

There is a paucity of data exploring the role of prophylactic TIPS in patients with portal hypertension undergoing non-hepatic surgery [170].

## Conclusions

Portal hypertension represents a serious, non-neoplastic sequelae of cirrhosis with high-risk complications. This consensus statement has been developed based on a systematic literature review and broad input from a diverse range of stakeholders and experts. It aims to equip both the referring team and multidisciplinary teams at TIPS centers with an in-depth understanding of the use of TIPS. It also aims to address the lack of dedicated Australian guidance on TIPS referral pathways and patient selection, taking into consideration our rapidly evolving knowledge about TIPS.

There remain numerous knowledge gaps and areas that warrant further research. Research on TIPS should focus on several key areas, including improving procedural technology and techniques, exploring new indications, refining patient selection criteria, developing strategies to manage complications, studying long-term outcomes to enhance patient survival and quality of life, and further developing services in Australia. Further research is also needed to understand the clinical effectiveness and utility of TIPS for rare indications. It is imperative to investigate the contextual factors that may limit access to and widespread use of TIPS in Australia. Research should focus on improving access to TIPS for Indigenous Australians and remote communities and identifying barriers to TIPS uptake. It is essential to recognize the unique cultural contexts of Aboriginal and Torres Strait Islander peoples and communities when conducting research and implementing health interventions. Adherence to the NHMRC *Ethical conduct in research with Aboriginal and Torres Strait Islander peoples and communities’* guidelines will be fundamental to ensuring best practice in working with Aboriginal and Torres Strait Islander peoples. These guidelines emphasize respect for cultural identity, community consultation and consent, reciprocity, collaboration and cultural safety.

Finally, research will be needed to investigate how widely these consensus recommendations are adopted in clinical practice. Ultimately, adherence to the best practice recommendations of this consensus statement may lead to system-level improvements in quality of life and outcomes and reduce variation in care.

## Data Availability

Not applicable.

## Abbreviations

ACC: American College of Cardiology
AGREE: Appraisal of Guidelines for Research and Evaluation
AHA: American Heart Association
BCS: Budd–Chiari syndrome
BOTEM: Bonn TIPS early mortality
CLIF-C ACLF: Chronic liver failure consortium acute-on-chronic liver failure
DIPS: Direct intrahepatic portacaval shunt
ePTFE: Expanded polytetrafluoroethylene
FIPS: Freiburg index of post-TIPS survival
GESA: Gastroenterological Society of Australia
GOV1: Gastro-esophageal varices type 1
GOV2: Gastro-esophageal varices type 2
GRADE: Grading of Recommendations Assessment, Development and Evaluation
HRS: Hepatorenal syndrome
IGV1: Isolated gastric varices type 1
INCPH: Idiopathic non-cirrhotic portal hypertension
IRSA: Interventional Radiology Society of Australasia
IVC: Inferior vena cava
LVP: Large-volume paracentesis
MELD: Model for end-stage liver disease
m-Delphi: Modified Delphi
MOTS: Modified TIPS score
MRI: Magnetic resonance imaging
PALBI: Platelet–albumin–bilirubin
PSPG: Portosystemic pressure gradient
PVT: Portal vein thrombosis
RVSP: Right ventricular systolic pressure
TAPSE: Tricuspid annular plane systolic excursion
TIPS: Transjugular intrahepatic portosystemic shunt
